# Compact Viscometer Prototype for Remote In Situ Analysis of Sludge

**DOI:** 10.3390/s19153299

**Published:** 2019-07-26

**Authors:** Tomas Fried, David Cheneler, Stephen D. Monk, C. James Taylor, Jonathan M. Dodds

**Affiliations:** 1 Engineering Department, Lancaster University, Lancaster LA1 4YW, UK; 2 National Nuclear Laboratory, Workington CA14 3YQ, UK

**Keywords:** rheology, nuclear decommissioning, rapid prototyping

## Abstract

On the Sellafield site there are several legacy storage tanks and silos containing sludge of uncertain properties. While there are efforts to determine the chemical and radiological properties of the sludge, to clean out and decommission these vessels, the physical properties need to be ascertained as well. Shear behaviour, density and temperature are the key parameters to be understood before decommissioning activities commence. However, limited access, the congested nature of the tanks and presence of radioactive, hazardous substances severely limit sampling and usage of sophisticated characterisation devices within these tanks and therefore, these properties remain uncertain. This paper describes the development of a cheap, compact, and robust device to analyse the rheological properties of sludge, without the need to extract materials from the site in order to be analysed. Analysis of a sludge test material has been performed to create a suitable benchmark material for the rheological measurements with the prototype. Development of the device is being undertaken with commercial off the shelf (COTS) components and modern rapid prototyping techniques. Using these techniques, an initial prototype for measuring shear parameters of sludge has been developed, using a micro-controller for remote control and data gathering. The device is also compact enough to fit through a 75 mm opening, maximising deployment capabilities.

## 1. Introduction

The landscape of the nuclear industry is changing nuclear power plants, and as reprocessing facilities approach the end of their planned lifespan, decommissioning has become a new focal point for the nuclear industry. This is reflected in academic research as well, with research moving from reprocessing operations to decommissioning [1]. An example of a decommissioning facility in the United Kingdom is the Sellafield site. Once the home of the first commercial nuclear reactor producing electricity on an industrial scale, the nuclear fuel reprocessing capability is approaching the end of its life cycle with the Thermal Oxide Reprocessing Plant (Thorp) facility stopping shearing in 2018 and Magnox reprocessing will end in 2020 [2,3].

Decommissioning of nuclear facilities requires a post-operational cleanout of active and hazardous materials. Using the Sellafield site as an example, there are a number of legacy tanks, silos and other containers with nuclear waste, often in the form of suspension, that will need to be cleaned out as part of the decommissioning plans. However, to accomplish this, analysis of remnant material must be performed to determine and minimise the risks, waste packages and ascertain the appropriate processes. Due to the hazardous nature of nuclear materials, this often has to be done remotely. Remote, in situ characterisation is, therefore, one of the key aspects of nuclear decommissioning [4].

The current strategy for analysis of remnant materials mainly focuses on visual inspection, radiological measurements and chemical composition analysis. Radiological measurements vary from using scintillators in storage tanks to determine the activity of remnant materials, pipe crawling robots assessing the contamination of pipework or autonomous platforms for analysis of floor contamination [5,6,7,8]. Current academic research focuses on imaging systems and the deployment of these in challenging environments. Advances in chemical composition analysis in nuclear facilities have introduced laser-induced breakdown spectroscopy (LIBS) as one potential method of remote analysis [9,10]. Visual inspection aids with assessing structures, waste locations and remnant material quantities in locations inaccessible to human workers. Most recently, advances in 3D scanning technologies (such as Light Detection and Ranging, or LiDAR) have been the focal point of research for visualizing remote areas. Other areas of research interest focus on the deployment platforms for both traditional camera systems and LiDAR in remote areas, whether through limited openings or underwater [11].

Currently used methods to ascertain rheological parameters of materials are predominantly rotational and oscillation rheometry using various geometries, either with samples or on-line [12,13,14]. Squeeze flow and capillary rheometry is frequently used in industries other than nuclear and academic research has been exploring these areas, too [15,16,17]. However, all of these methods utilise large, expensive equipment and development focus on increasing the precision and measurement range of devices.

In situ rheology in hazardous environments is, however, an unexplored research area. Research has been limited to test materials, or it relies on collecting samples from storage containers [18,19,20]. Novel methods for rheological measurements that have been developed are focused on miniaturisation and small sample methods, such as quartz crystal microbalance, but these are still in early research stages [21].

This paper presents work undertaken to develop a compact, cheap and robust device for shear behaviour analysis and its initial validation in a non-active laboratory environment. The aims are to propose a novel prototype device; to manufacture this device using rapid prototyping methods and commercial off the shelf (COTS) components; to calibrate the new device using industrial standard materials and to validate its performance at a laboratory bench scale. Section 2 provides a summary of materials and conventional instruments that have been used for analysis and manufacturing. Section 3 provides a description of the designed device, the tests performed, and the results of these tests. Finally, the discussion and conclusions are presented in Section 4.

## 2. Materials and Methods

A Bohlin CVO100 (Malvern Panalytical Ltd, Malvern, UK) was used as the baseline, commercially available, benchtop rheometer for the development of the device. All measurements made with the commercial rheometer were logarithmically spaced shear rate ramps from 0.1 to 100 s^−1^ at ambient temperatures between 21.5 and 22.5 °C, with a DIN standard V25 vane and cup and C25 bob and cup configurations. All measurements consisted of five measurement runs, and the results presented are averages of those five runs.

A silicone viscosity standard oil (Paragon Scientific Ltd, Merseyside, UK, VIS-RT1K-600) has been used as the calibration material. It is an industry-standard material used for calibrating rheometers and viscometers.

Two test materials were chosen to validate the performance of the device when measuring particulate suspensions. The first material was titanium dioxide (anatase, Sigma-Aldrich, Louis, MO, USA, 248576). This is an example of a lower viscosity, non-Newtonian particulate suspension. The TiO_2_ was supplied as a powder, which was dried and subsequently dispersed in de-ionized water at a volume fraction of ϕ = 0.44. TiO_2_ has been historically used as a very safe test material in the nuclear industry [20]. Although it has since been replaced with other test materials, it remains an appropriate test material for the early stages of device development.

The second material was zirconium-molybdate (ZM) suspended in 0.5 M nitric acid. The sample was synthesised by Johnson Matthey and supplied as a suspension. Some of the supernate was decanted, and a sample was dried to ascertain the volume fraction of the suspension. ZM is an example of a higher viscosity test material. It is the most novel simulant being used in the nuclear industry [22].

**Figure 1:**
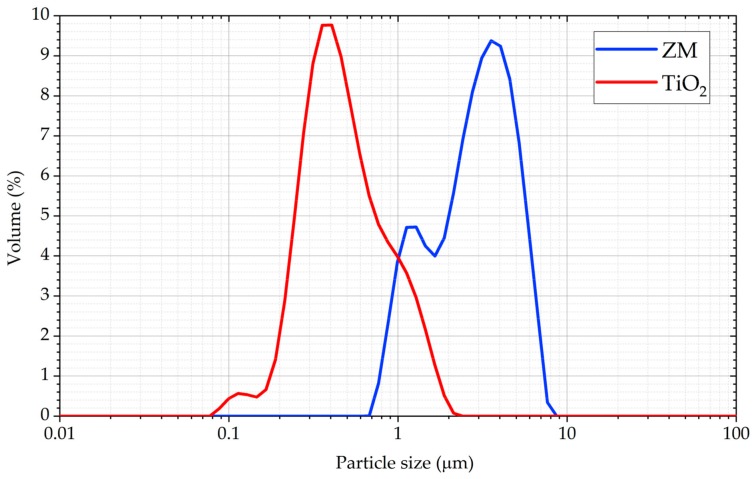
Particle size distribution (PSD) of the titanium dioxide and zirconium molybdate samples used in this research.

A Malvern Mastersizer 3000 (Malvern Panalytical Ltd, Malvern, UK) particle characterisation system with a wet dispersion unit was used to ascertain the particle size distribution (PSD) of the two test materials, as shown in Figure 1 Both materials were dispersed in de-ionized water (refractive index 1.33). TiO_2_ was stirred at 1800 rpm and measured at approximately 3.7% laser obscuration, using a refractive index 2.493 and an absorption index 1. ZM was stirred at 2400 rpm and measured at approximately 30.7% laser obscuration, using a refractive index 1.19 and an absorption index 0.

A majority of the structural components and other parts were manufactured using a fused deposition modelling 3D printer (Ultimaker 3 extended, Ultimaker, Utrecht, The Netherlands). Two different materials were utilised as follows. Linkages and outer casings not coming into contact with chemical substances were made using Ultimaker PLA. PLA is a biodegradable, cheap and straightforward to use material offering high stiffness. It is the most appropriate material for rapidly prototyping initial development prototypes, as required for the present work. Measurement geometries and their corresponding outer cups were manufactured with XSTRAND^TM^ GF30-PP—glass fibre reinforced polypropylene (Owens Corning, Toledo, OH, USA). This material offers much higher chemical resistance and thermal stability compared to PLA, making it suited for use with chemically aggressive substances, such as ZM in nitric acid. Table 1 compares the mechanical and other properties of these materials.

## 3. Device Development and Results

The present section describes the new device, its calibration and evaluation results.

### 3.1. Description of Designed Device

The device is a rotational viscometer, using bob and vane geometries with outer cups. It uses a spring-loaded mechanism to measure the reaction torque from the analysed substance as a function of the deflection of the drive system. 

**Figure 2:**
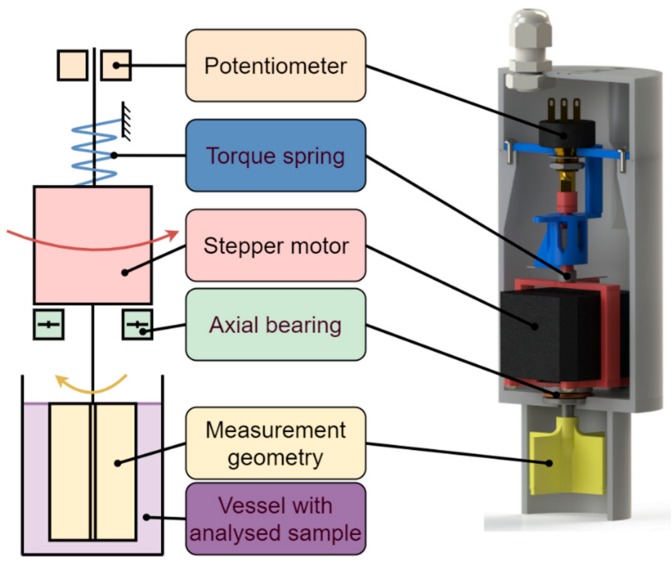
Functional schematic and a render of the prototype.

**Figure 3:**
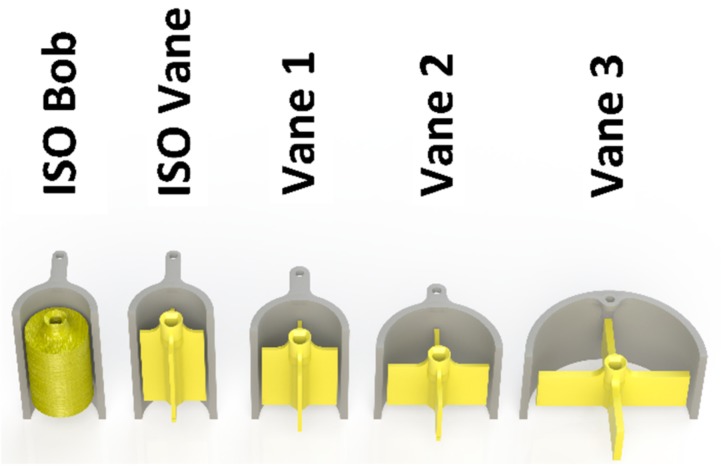
Cutaway render of all the geometries used with the prototype.

Figure 2 illustrates the mechanism and the major parts used in it. A stepper motor (RS Components Ltd, Corby, UK, RS PRO 535-0372) drives a measurement geometry (described further below and pictured in Figure 3) with a constant rotational velocity in the substance it is analysing. When the measurement geometry shears the sample, the reaction torque turns the entire drive assembly in the opposite direction, as the motor is held in place by a bearing that allows it to rotate around the same axis as the measurement geometry. A spring connecting the drive assembly to the stationary frame ensures that the mechanism does not spin continuously, i.e., it only deflects proportionally to the shear stress of the material that the measurement geometry shears.

Two of the geometries and their respective cups used with the device are designed based on the dimensions proposed by the ISO 3219 standard, widely used in the design of conventional benchtop rheometers [25]. They are manufactured with the same dimensions as the V25 vane and C25 bob used on the Bohlin CVO100 rheometer. The device, however, diverges from the ISO standard in two main regards. One, since it is deployed by inserting it in a sample, the cup is open on the bottom. This allows the material to enter the cup and surround the measurement geometry as the device is inserted into the material. Secondly, the immersion height is not controlled by sampling but by deploying the device. In the experiments described below, the material height is controlled in the test material vessel.

In potential deployment scenarios, the material height may not be sufficient to fully immerse long geometries. For this reason, three other geometries have been designed and used, diverging from the ISO standard. Another consideration is preventing the slip of the geometry when it rotates in the material. Vanes are often used with suspensions and higher viscosity samples, as they slip less than bobs when shearing these samples. Finally, inserting the geometry itself affects the structure of the sample. Vanes penetrate samples much easier than bobs. Based on all of these considerations, all three non-standard geometries (i.e., Vane 1, 2 and 3 in Figure 3) take the form of four-bladed vanes.

As described in the ISO standard, the representative shear rate in the middle of the gap between the geometry and the cup ${\dot{\gamma }}_{rep}$ is determined as shown in Equation (1).

(1) $${\dot{\gamma }}_{rep}=\Omega \cdot \frac{1+{\left(\frac{r_{e}}{r_{i}}\right)}^{2}}{{\left(\frac{r_{e}}{r_{i}}\right)}^{2}-1},$$

where *Ω* is the angular velocity of the geometry, *r_e_* is the radius of the outer cylinder (cup) and *r_i_* is the radius of the inner cylinder (geometry). It is apparent that the shear rate is dependent on the ratio of the dimensions of the geometry and the cup, and thus, the ratio has been maintained constant with all the geometries. To accommodate potential low material heights, the non-standard vanes have been designed to be shorter and thus require less material to be fully immersed. To ensure that the torque is not below the detection threshold when performing the measurement, the radius of the geometry has been increased in comparison with the ISO geometries.

A summary of the geometry dimensions that have been manufactured and used with the prototype is outlined in Table 2, while the geometry and cup configuration is illustrated in Figure 3.

The outer case and internal linkages were 3D printed using a fused deposition modelling printer with Ultimaker PLA. The bob and vane geometries and the outer cups were 3D printed with XSTRAND^TM^ GF30-PP. Overall, the largest configuration (ISO vane or bob) of the device is 219 mm long (i.e., edge of the cable gland to end of the geometry cup) and 75 mm in diameter at the widest point.

**Figure 4:**
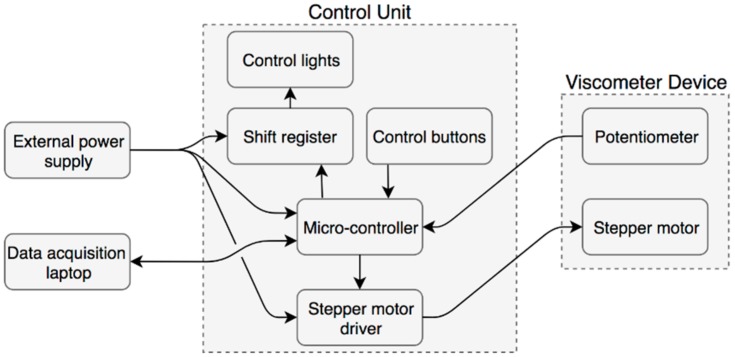
Schematic of the electronic components used with the prototype.

The prototype was designed to remove as many sensitive electronic components from the deployment environment. Hence, the electronic circuitry consists of two main assemblies (Figure 4).

**Figure 5:**
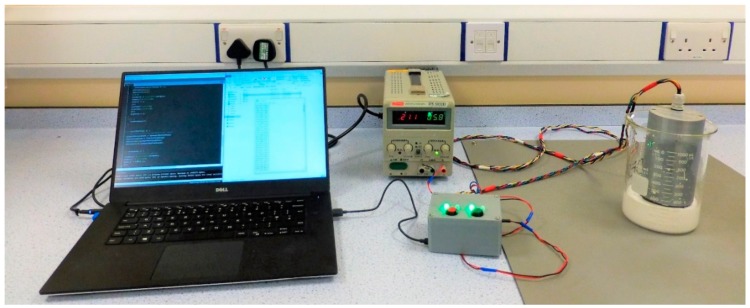
Experimental setup with the prototype. Devices from left to right: laptop, external power supply, control unit, viscometer prototype device in a beaker immersed in a sample of TiO_2_.

The Control Unit houses all the data acquisition and control electronics necessary to drive the mechanism and read the data from an on-board sensor. All of the components used are COTS products. A Cortex-M4F (180 Mhz rated core speed) based microcontroller was selected as it supports the C++ based programming language, has integrated digital-to-analogue conversion (DAC) functionality and 16-bit resolution analogue-to-digital conversion (ADC) functionality. It controls the stepper motor through a Pololu DRV8834 stepper motor driver (Pololu Corporation, Las Vegas, NV, USA) and reads the output of the Bourns 6630S0D-C28-A103 continuous turn potentiometer (Bourns, Riverside, CA, USA) in the viscometer device. Furthermore, it reads two buttons used to start and stop the pre-tensioning and measurement procedures and controls the 74HC595 shift register used to display the current state of the prototype through 4 LED indicators on the top panel of the Control Unit. All of the Control Unit components are in a PLA housing. The overall dimensions of the housing are 100 mm × 80 mm × 53 mm. All of the units can be seen in Figure 5.

The prototype is programmed with two procedures. One is to zero the mechanism to establish the origin of the measurement. This procedure runs the geometry in the opposite direction of the measurement rotation in a ramp-up of rotational velocity, which deflects the mechanism. When the mechanism deflects beyond a pre-programmed value, the program switches to a ramp down to allow the mechanism to settle, in what will be used as a zero position. This procedure serves to eliminate all potential deflection and tensioning of the mechanism during deployment.

The second procedure is the measurement itself. The prototype is programmed to supply a ramp-up of rotational velocities in integer values. The stepper motor supplies this velocity for a total of 12 s, measuring the deflection of the mechanism in the last six seconds to allow the mechanism to fully deflect before the measurement. In those six seconds, the microcontroller reads and saves the value from the potentiometer every 400 ms, logs the time since it started the measurement procedure and saves the rotational velocity value it is currently supplying to the geometry.

### 3.2. Calibration Procedure

**Figure 6:**
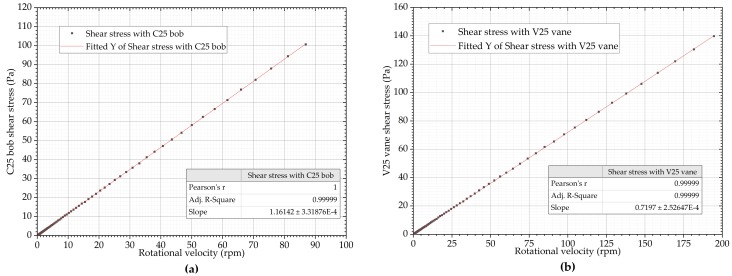
Shear stress as a function of the rotational velocity of silicone viscosity standard oil on a commercial rheometer with: (**a**) C25 bob and cup configuration; (**b**) V25 vane and cup configuration.

A silicone viscosity standard oil (Paragon Scientific VIS-RT1K-600) was used as the calibration material. A baseline measurement was performed using a Bohlin CVO100 rheometer with this oil, using a logarithmically spaced shear rate ramp from 0.1 to 100 s^−1^ over 30 s, with a DIN standard V25 vane a C25 bob and cup configurations. Five runs were made with both geometries, with the averaged shear stress results and the linear fits of data presented in Figure 6. The results are plotted as a function of rotational velocity since the prototype device supplies rotational velocity in revolutions per minute.

**Figure 7:**
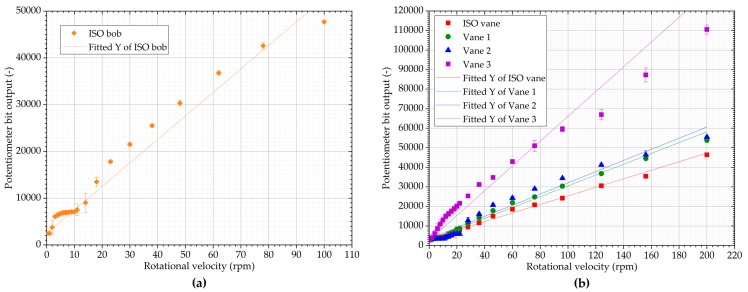
Potentiometer bit output as a function of revolutions per minute of silicone viscosity standard oil with the prototype with (**a**) C25 bob and cup configuration; (**b**) Selection of vanes.

The same calibration oil was then used with the prototype and all of its geometries. The prototype supplied rotational velocities in the range of 1 to 100 rpm with the bob and 2 to 200 rpm with the vanes (corresponding to roughly 1 to 100 s^−1^ on the commercial rheometer). The spacing is approximately logarithmic, limited by the fact that the prototype can supply only integer values of revolutions per minute. Five runs with the standard oil were performed for each geometry and the raw data were fitted with linear functions, as illustrated by Figure 7.

The raw data collected with the potentiometer in the prototype indicates the torque applied to the vane. The raw data were fitted with a linear function and, using data collected with the conventional rheometer, the relationship between the raw data and expected shear stress based on the conventional rheometer measurement was determined. Using this approach, the conversion constants between raw data and shear stress were obtained for each geometry. Summary of calibration data can be seen in Table 3.

**Figure 8:**
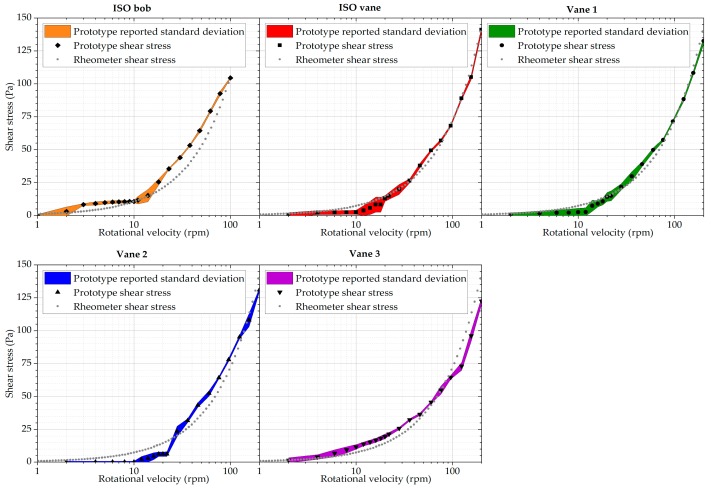
Shear stress results obtained with all five prototype geometries during the calibration procedure, in comparison to a conventional rheometer.

Figure 8 summarises the calibration results for all five geometries. Vane 3 offers the best low shear stress detection rate. However, the linearity is limited in the higher shear stress regions. Smaller vanes, namely the ISO and Vane 1 geometries exhibit better repeatability and linearity in higher rotational velocities, but as the torques are lower, low shear stress detection is relatively poor. The bob geometry and Vane 2 exhibit the poorest results overall. With the bob geometry, this can be attributed to the potentially highest sensitivity to axial misalignment of the bob and the cup, with the results being affected by the uneven gap between the bob and the cup.

**Figure 9:**
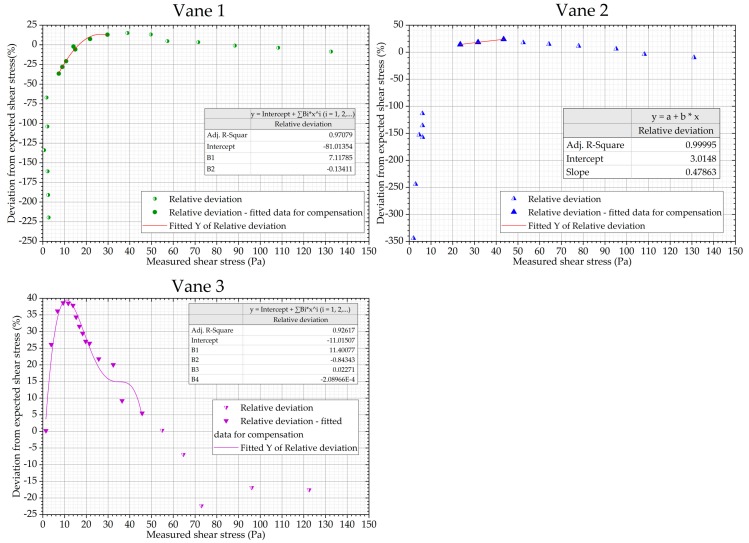
Non-linearity of the prototype with Vanes 1, 2 (first five values are off the scale and not used in calculations) and Vane 3 using a silicone viscosity standard oil and fitting functions for compensation.

All the geometries exhibit repeatable, systematic deviations from the expected linear response to the silicone oil. Therefore, a compensation procedure was created to remove the nonlinearities. For all the geometries, the relative deviation from the expected value of shear stress was plotted against the shear stress measured with the prototype. Polynomial functions were fitted to the values of the deviation as a function of the measured shear stress, as illustrated in Figure 9. To ensure a good fit using low order functions, only data in the range necessary to be compensated were fitted. These functions are subsequently used to determine the compensation value for the measured results. Actual reported shear stress values are, therefore, defined as the measured shear stress minus the value of the polynomial function at that measured shear stress.

It is important to note that the results obtained with this procedure must be used carefully and that the compensation should only be used within the range of the fitted data, see Figure 9. The results discussed below illustrate instances in which the procedure appears to be appropriate or not.

One aspect of using vanes in rotational rheology that needs to be considered when interpreting the results are secondary flows and vortices appearing in the sample. Research on quantifying the onset of secondary flows using vanes is limited and only applied to one phase materials. Onset of Taylor vortices can be described using the Taylor number. For concentric cylinders, with the outer cylinder stationary and the inner cylinder rotating, in the middle of the gap between the geometries, the Taylor number *T_a_* is often calculated as shown in Equation (2) [26].

(2) $$Ta=\frac{r_{i}{\left(r_{e}-r_{i}\right)}^{3}\Omega_{i}^{2}}{\nu^{2}},$$

where *ν* is the kinematic viscosity of the sample.

The onset of turbulent flow in the same setup as considered in the calculation of the Taylor number is referred to as the rotational Reynolds number and can be calculated as illustrated in Equation (3).

(3) $$Re=\frac{r_{i}\left(r_{e}-r_{i}\right)\Omega_{i}}{\nu }.$$

**Figure 10:**
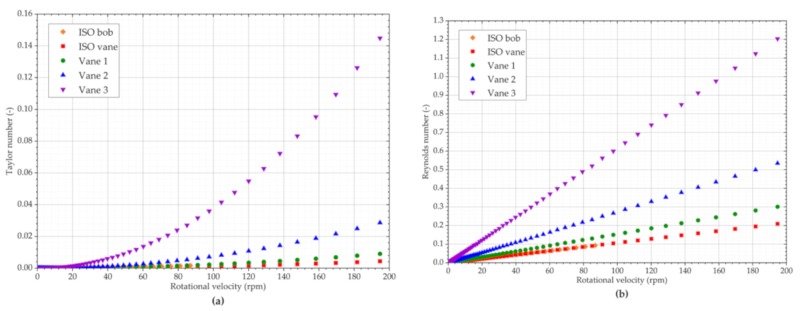
Potential indicators of secondary effects when measuring standard silicone oil: (**a**) Taylor number for all geometries; (**b**) Reynolds number for all geometries.

It is important to note that these parameters have been validated and used with concentric cylinders and their validity for vanes with particulate suspensions has not been investigated in the literature to date. Rotational rheology using vanes assumes the material shears along the blades of the vanes, and thus vanes are often described using the same principles as concentric cylinders. Current research and rheology laboratory equipment suppliers suggest that vanes are more susceptible to both turbulent flows and Taylor vortices. However, it is apparent from Equations (2) and (3) that it is assumed here that both vanes and bob geometries exhibit the same behaviour. Both Taylor and Reynolds numbers have been calculated using the viscosity and angular velocity determined on the rheometer with the calibration silicone oil and the dimensions of the geometries used with the prototype. The results are illustrated in Figure 10.

### 3.3. Measurements with TiO_2_ Suspension

**Figure 11:**
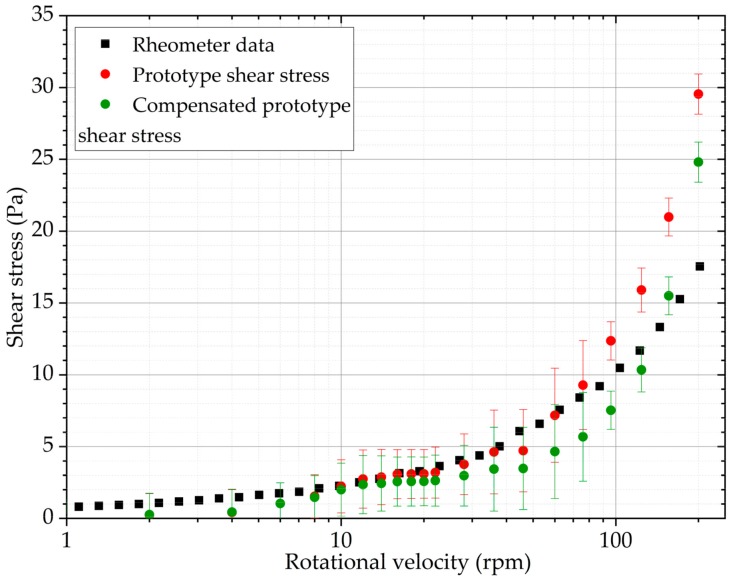
Shear stress results obtained with a rheometer, the prototype (using vane 3) and compensated prototype values with titanium dioxide suspension.

The titanium dioxide suspension is low viscosity, and the shear stress is below the detection level of most geometries, with the exception of Vane 3. Using this geometry, as shown by Figure 11, the uncompensated data aligns with the expected values. However, at approximately 80 rpm, the slope of the curve changes and for high shear rate regions the prototype starts to report values higher than expected. The most likely explanation is secondary flows starting to appear in the analysed material. It is apparent that the compensation procedure is not appropriate for low viscosity sample such as used here, as it causes under-reporting.

**Figure 12:**
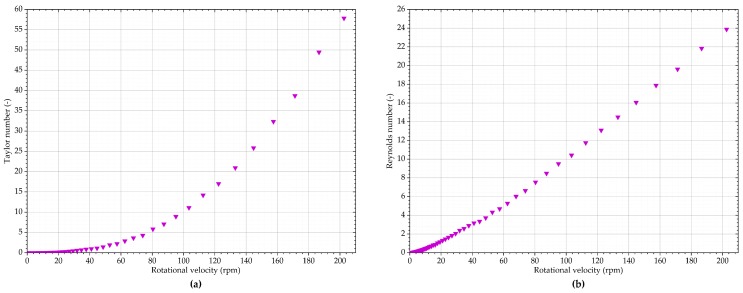
Potential indicators of secondary effects when measuring titanium dioxide suspension with Vane 3: (**a**) Taylor number; (**b**) rotational Reynolds number.

The Taylor and Reynolds numbers calculated using the rheometer collected data, but with the dimensions of Vane 3, are illustrated by Figure 12. Secondary flow effects appear to start at around 80 rpm, corresponding to *Ta* = 6 and *Re* = 7.5. These values are low, compared to reported thresholds indicating the onset of secondary effects in current literature.

### 3.4. ZM Suspension

**Figure 13:**
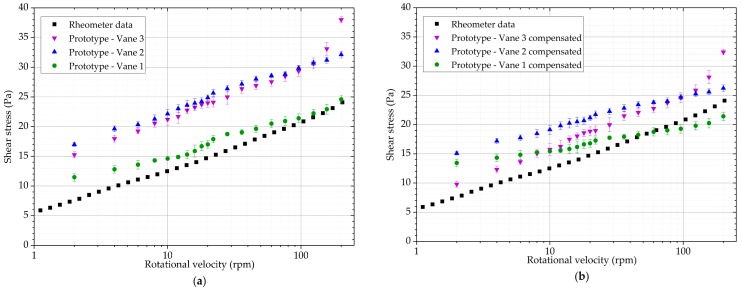
Shear stress results obtained with the rheometer and the prototype with Vanes 1, 2 and 3: (**a**) Measured values; (**b**) Compensated values.

ZM suspension has a much higher viscosity in the low rotational velocity regions. It is also demonstrably shear thinning. It is apparent from Figure 13 that the values obtained with Vane 2 and Vane 3 are higher than expected. With Vane 2, this can be attributed to the relatively poor calibration results. With Vane 3, some over-reporting is expected due to the non-linearity of the response of the prototype. Vane 1 appears to offer the best approximation of the results without compensating the results. All three geometries report shear thinning behaviour.

The compensation procedure does not seem appropriate for Vane 1 in this case, as it changes the slope of the curve. Vane 2 is affected similarly. However, the values obtained with the prototype are still higher than expected across the measurement range. The procedure works well for Vane 3, as the slope of the data is still the same, but results are much closer to the expected values.

**Figure 14:**
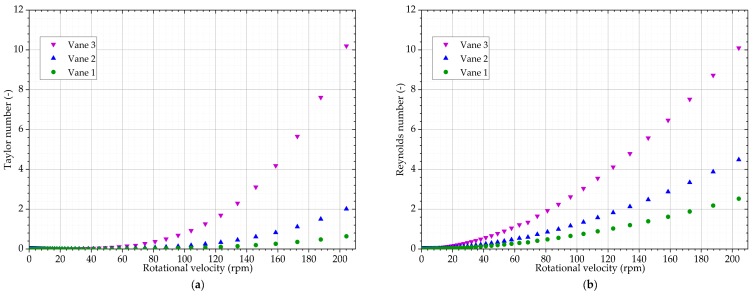
Potential indicators of secondary effects when measuring zirconium molybdate suspension with Vane 1, 2 and 3: (**a**) Taylor number; (**b**) rotational Reynolds number.

The potential onset of secondary flow seems to be delayed as opposed to the lower viscosity sample. This is consistent with the calculations of the Taylor and Reynolds numbers, as increased viscosity is expected to delay the onset of secondary flows. The secondary flow effects appear only with Vane 3, from approximately 150 rpm, corresponding to *Ta* = 3.5 and *Re* = 6, as illustrated in Figure 14. These values are similar to those obtained with titanium dioxide suspension.

## 4. Discussion and Conclusions

This paper has described the development of a relatively compact and robust device to analyse the rheological properties of sludge without the need to sample materials. Analysis of a sludge test material has been performed to create a suitable benchmark material for the rheological measurements with the prototype.

The results presented in this paper show the advantages and disadvantages of the proposed mechanism and measurement geometries to obtain shear behaviour data of suspensions. The prototype device and all necessary electronics are all either COTS components, or 3D printed, and the total price of the prototype is less than £250. The device also only requires freeware programs to run and transfer data to a PC.

The calibration procedure shows the non-linearity of all geometries. Non-standard geometries offer responses that can be compared with ISO/DIN standard geometries. Larger geometries offer beneficial low shear stress responses, making them suitable for low viscosity samples and potentially for yield stress analysis. The prototype is able to reproduce measurements of lower viscosity suspension samples using the calibration and compensation methods proposed in this article.

No secondary flow effects were observed with any of the geometries using the 1Pa·s silicone standard oil. Potential secondary flow effects were observed in the suspensions at angular velocities over approximately 100 rpm. However, as the tool is being developed for ascertaining low shear rate behaviour, these effects are not prohibitive. Further investigation would be necessary to quantify the onset of these effects. The present results indicate that secondary flows might start appearing at very low Taylor and rotational Reynolds numbers <10.

Vane 3 offers the most comparable results, with a compensation procedure appropriate for the zirconium molybdate sample. Shear-thinning behaviour was correctly reproduced with all three vanes on the ZM sample, and the compensation procedure lowers the deviation from the expected results considerably. A more advanced mathematical model could further improve the compensation procedure. The performance indicates that the prototype would be suitable for deployment, especially considering the low manufacturing costs and the potential to reuse components. Vane 3 is the obvious candidate as it requires the least material to be present in the deployment area and provides the most accurate and consistent results. However, it also requires careful interpretation of high shear rate results due to the possibility of the onset of secondary flows.

The device is compact enough to fit through a 75 mm opening, maximising deployment capabilities. The deployment of the device can be accomplished in various ways, depending on the deployment area. The first option is manual deployment, which would be suitable for smaller containers that can be approached by operators. The device would simply be lowered into the container using an extension rod. The second option relates to the assessment of settled bed behaviour in larger tanks. Multiple underwater remotely operated vehicles (ROVs) have been designed and deployed on various sites and are capable of carrying payloads such as the proposed device [27]. The prototype could be carried and detached from the carrying platform when it reaches the required position. Lastly, for remote tanks, pipe crawler and tracked robots have been used to carry sensors and other payloads and these platforms could deploy the device in harder to access areas [28,29]. In all instances, the tether would serve not only a data transferring function but also as a means of recovery of the device from these areas. The device is light (0.5 kg in the laboratory scale configuration), and the casing is robust enough to support a recovery procedure using a tether. The simple assembly would make it easy to reuse the internal components if necessary and decontaminate the outer casing, cups and geometries after use. If the device cannot be decontaminated, the low price supports the case to use the device only once before disposal.

Potential future development of the device could include lowering friction in the mechanism, which would improve low shear stress detection and repeatability of the measurements. A more complex mathematical model for the non-linearity compensation would be beneficial for the interpretation of the results. Lastly, understanding the onset of secondary flows in various particulate suspensions would be necessary to increase the operating range of the instrument.

Overall, the precision and performance of the proposed mechanism makes it suitable for preliminary analysis of sludge in situ. The capability of the current stage of the prototype would make it useful for determining the rough properties of the materials, to inform further decisions as necessary in the upcoming steps of Post Operational Clean Out (POCO).

## Figures and Tables

**Table 1:** Comparison of the parameters of Ultimaker PLA and XSTRAND^TM^ GF30-PP 3D printing filaments [23,24].

	Ultimaker PLA	XSTRAND^TM^ GF30-PP
Tensile strength at yield (MPa)	49.5	60.0
Flexural strength at yield (MPa)	103.0	83.0
Maximum usable temperature (°C)	50	120
Chemically resistant	No	Yes

**Table 2:** Comparison of the dimensions of geometries used with the device.

	ISO Bob	ISO Vane	Vane 1	Vane 2	Vane 3
Cup Radius *r_e_* (mm)	13.75	13.75	16.50	22.00	33.00
Geometry Radius *r_i_* (mm)	12.50	12.50	15.00	20.00	30.00
Geometry Length *L* (mm)	37.50	37.50	30.00	20.00	15.00
*L*/*r_i_* (-)	3.00	3.00	2.00	1.00	0.50
Necessary Sample Height (mm)	52.50	52.50	42.50	32.50	27.50

**Table 3:** Summary of calibration data.

	ISO Bob	ISO Vane	Vane 1	Vane 2	Vane 3
R2 of raw data fit (-)	0.9826	0.9970	0.9968	0.9786	0.9793
Slope of prototype raw data fit (-)	502.73	218.72	276.74	285.94	633.86
Slope of commercial rheometer data fit (Pa)	1.1614	0.7197			
